# Growth Differentiation Factor‐15 Deficiency Inhibits Atherosclerosis Progression by Regulating Interleukin‐6–Dependent Inflammatory Response to Vascular Injury

**DOI:** 10.1161/JAHA.112.002550

**Published:** 2012-12-19

**Authors:** Gabriel A. Bonaterra, Stefanie Zügel, Joel Thogersen, Sabrina A. Walter, Uwe Haberkorn, Jens Strelau, Ralf Kinscherf

**Affiliations:** 1 Institute of Anatomy and Cell Biology, Department of Medical Cell Biology, University of Marburg, Marburg, Germany (G.A.B., S., S.A.W., R.K.); 2 Department of Anatomy and Cell Biology III, University of Heidelberg, Heidelberg, Germany (J.T., J.S.); 3 Department of Nuclear Medicine, University Hospital of Heidelberg, Heidelberg, Germany (U.H.)

**Keywords:** atherosclerosis, GDF‐15, inflammation, interleukins

## Abstract

**Background:**

Growth differentiation factor (GDF)‐15 is a distant and divergent member of the transforming growth factor‐β superfamily (TGF‐β) . There is growing evidence indicating the involvement of GDF‐15 in various pathologies. Expression of GDF‐15 is induced under conditions of inflammation and increased GDF‐15 serum levels are suggested as a risk factor for cardiovascular diseases.

**Methods and Results:**

We show here that GDF‐15 and proinflammatory cytokine interleukin (IL)‐6 levels are highly increased (5‐fold) in cultured oxidized low‐density lipoproteins–stimulated peritoneal macrophages derived from GDF‐15^+/+^/apolipoprotein (apo) E^−/−^, mice. Notably, IL‐6 induction on oxidized low‐density lipoproteins stimulation is completely abolished in the absence of GDF‐15. Consistent with our in vitro data GDF‐15 mRNA expression and protein levels are upregulated (2.5‐ to 6‐fold) in the atherosclerotic vessel wall of GDF‐15^+/+^/apoE^−/−^ mice after a cholesterol‐enriched diet. GDF‐15 deficiency inhibits lumen stenosis (52%) and ^18^FDG uptake (34%) in the aortic arch despite increased serum triglyceride/cholesterol levels and elevated body weight. Immunohistomorphometric investigations of atherosclerotic lesions reveal a decreased percentage of inflammatory CD11b^+^ (57%) or IL‐6^+^, leukocytes, and apoptotic cells (74%) after 20 weeks. However, the total number of macrophages and cell density in atherosclerotic lesions of the innominate artery are increased in GDF‐15^−/−^/apoE^−/−^ mice.

**Conclusions:**

Our data suggest that GDF‐15 is involved in orchestrating atherosclerotic lesion progression by regulating apoptotic cell death and IL‐6–dependent inflammatory responses to vascular injury.

## Introduction

Atherosclerosis is a chronic inflammatory process characterized by the accumulation of lipids, inflammatory cells and fibrous elements in the arterial wall. Increased serum cholesterol levels and especially “bad” low‐density lipoprotein (LDL) cholesterol in the form of oxidized low‐density lipoproteins (oxLDL) are believed to play a key role during all stages of the disease by regulating the expression of chemokines and proinflammatory cytokines. Despite intense research efforts, the precise mechanisms leading to development and progression of atherosclerotic lesions remain largely unknown. This also applies to the still incomplete picture of the lesion‐mediated regulation of growth factors and, in particular, their functions for lesion development. In this context, growth‐differentiation factor (GDF)‐15 has been suggested as a biomarker for cardiovascular diseases.^1^ GDF‐15 is a distant and divergent member of the transforming growth factor (TGF)‐β superfamily, which does not belong to one of the TGF‐β subfamilies.^2–5^ GDF‐15 has been cloned and characterized in our and several other laboratories.^3–5^ The protein is identical to (1) macrophage‐inhibitory cytokine‐1, (2) NSAID‐activated gene‐1, (3) placental bone morphogenetic protein—which all have been found in macrophages (MФ)—and to some other published sequences.^6–8^ We and others have shown that GDF‐15 is a lesion‐induced factor implicated in a number of pathophysiological processes including inflammation, chronic vascular diseases, cancer, ischemia, and atherosclerosis.^1,9–12^ Moreover, circulating levels of GDF‐15 are suggested as a prognostic marker to improve risk stratification of patients with acute coronary syndrome, which may also help in selection of patients who benefit from invasive therapy.^13–14^ However, the cellular sources, upstream regulators, and functional effects of GDF‐15 in the cardiovascular system have not been elucidated.^11^ A major still unresolved problem is the identification of GDF‐15 specific receptor(s) and signalling pathways. In this context, several studies have suggested that GDF‐15 signal transduction is mediated through classic TGF‐β receptors and according downstream (ie, Smad) cascades.^15–16^ However, studies in our laboratory so far failed to confirm GDF‐15 signaling via TGF‐β (or glial cell line–derived neurotrophic factor [GDNF]) receptors (Strelau, 2004, unpublished results).

Development and progression of atherosclerotic plaques are driven by endothelial dysfunction, oxLDL deposition in the subendothelial space, and recruitment as well as attachment of inflammatory monocytes to the arterial vessel wall, their differentiation into MФ, and subsequent transformation into cholesterol‐laden foam cells in the subendothelial space. We have previously reported that GDF‐15 immunoreactivity is localized in MФ of human atherosclerotic carotid arteries and colocalizes with oxLDL, as well as apoptosis‐relevant proteins.^17^ Furthermore, we have shown that induction of apoptosis in cultured MФ (eg, by oxLDL) correlates with increased GDF‐15 expression.^17^ In addition, GDF‐15 expression is increased after stimulation by proinflammatory cytokines such as tumor necrosis factor (TNF)‐α, interleukin (IL)‐1β, or IL‐6 but not by interferon‐γ and lipopolysaccharide.^2^

We show here that GDF‐15 expression (RNA and protein) is highly upregulated in the atherosclerotic vessel wall. Consistently, lack of GDF‐15 revealed a significant long‐term reduction of atherosclerotic lesion formation. This effect was accompanied by decreased occurrence of inflammatory CD11b^+^ or IL‐6^+^ leukocytes, apoptotic cell death; however, an increased percentage of MФ and enhanced cell density in atherosclerotic lesions of the innominate artery (=brachiocephalic trunk) suggested that GDF‐15 is involved in orchestrating atherosclerotic lesion progression.

## Methods

### Animals

GDF‐15 knockout/lacZ knockin (GDF‐15^−/−^) mice^18^ were crossbred with apolipoprotein (apo)E knockout (apoE^−/−^) mice (Charles River, Sulzfeld, Germany). Male homozygous null and wild‐type (WT) mice were used for experiments. All animal experiments were approved by the Regierungspräsidium Karlsruhe and the local authorities at the University of Heidelberg and were done in compliance with the regulations for animal studies at the University of Heidelberg.

### Genotyping

Genomic DNA was isolated, according to the manufacturer's instructions (DNA Extraction Solution; Epicentre Biotechnologies, Madison, WI). Transgenic positive animals were identified by polymerase chain reaction (PCR) of genomic tail DNA, using intron‐spanning oligonucleotides (Table 1). PCR analysis showed representative single bands for GDF‐15 knockout (690 bp), GDF‐15 WT (320 bp), apoE knockout (245 bp), and apoE WT (155 bp; data not shown).

**Table 1.** Oligonucleotides Used for Genotyping Polymerase Chain Reaction

Primer Name	Sequence	Amplicon Length	Company
GDF‐15‐knockout			
NeoF1 forward	5′‐TCG CCT TCT TGA CGA GTT CT‐3′	690 bp	Metabion, Planegg‐Martinsried, Germany
R20 reverse	5′‐CCC AGT CTT GTA GAC AGA GCA A‐3′		Metabion, Planegg‐Martinsried, Germany
Wild‐type			
F26 forward	5′‐ATG CGC ACC CAA GAG ACT‐3′	320 bp	Metabion, Planegg‐Martinsried, Germany
R21 reverse	5′‐GGC CAC CAG GTC ATC ATA AG‐3′		Metabion, Planegg‐Martinsried, Germany
Apolipoprotein E knockout			
oIMR180 forward	5′‐GCC TAG CCG AGG GAG AGC CG‐3′	245 bp	Metabion, Planegg‐Martinsried, Germany
oIMR182 reverse	5′‐GCC GCC CCG ACT GCA TCT‐3′		Metabion, Planegg‐Martinsried, Germany
Wild‐type			
oIMR180 forward	5′‐GCC TAG CCG AGG GAG AGC CG‐3′	155 bp	Metabion, Planegg‐Martinsried, Germany
oIMR181 reverse	5′‐TGT GAC TTG GGA GCT CTG CAG C‐3′		Metabion, Planegg‐Martinsried, Germany

GDF‐15 indicates growth‐differentiation factor‐15.

### Lipoprotein Preparation

LDL was isolated from the plasma of normolipemic donors by differential ultracentrifugation as previously described^19^ and dialyzed against Dulbecco's PBS without Ca^2+^ and Mg^2+^ (PAA Laboratories GmbH, Pasching, Austria). Before oxidation, the concentration of native LDL (nLDL) was adjusted to 1.5 mg/mL in Dulbecco's PBS. LDL was oxidized with 50 μmol/L CuSO_4_ in DPBS (18 to 24 hours), whereas oxidation was stopped with 50 μmol/L EDTA in PBS. The electrophoretic mobility of oxLDL—in comparison with BSA and nLDL—was determined by agarose gel electrophoresis and visualized by staining with Coomassie Blue.

### Isolation of Peritoneal Macrophages and Real‐Time PCR (qRT‐PCR)

To obtain an increased number of peritoneal macrophages, mice were injected with 500 μL of pristane (Sigma–Aldrich, Steinheim, Germany) into the lower left quadrant of the abdominal cavity perpendicularly to the fold for no more than 0.5 cm of the needle. After 2 days, mice were killed by cervical dislocation and peritoneal cells were removed by peritoneal lavage with 6 mL of ice‐cold PBS buffer. Collected cells were washed twice with PBS and resuspended in RPMI medium containing 10% FBS, 1% penicillin‐streptomycin‐neomycin, and 1% glutamine. After 1 hour (37°C, 5% CO_2_), nonadherent cells were discarded. Adherent cells were incubated overnight before being exposed to native LDL 100 ng/mL, oxLDL 100 ng/mL, or medium alone for 12 hours. Subsequently, the cells were washed and extraction of total RNA was performed using TriFast solution (PeqLab, Erlangen, Germany) according to the manufacturer's standard protocol.

Tissue samples were transferred immediately after preparation into RNA‐later‐ICE (Ambion, Austin, TX) and homogenized in Precellys tubes with ceramic beads in a Precellys homogenizator (PeqLab). Total RNA extraction was performed according to the manufacturer's instructions (TriFast; PeqLab).

RNA concentration and purity were determined by A260 and A280 (A260/A280=1.7 to 2.0) measurements using a NanoDrop 8000 Spectrophotometer (Thermo Scientific, Schwerte, Germany). Total RNA integrity was confirmed using lab‐on‐a‐chip technology, using an RNA 6000 NanoChip kit on an Agilent 2100 Bioanalyzer (Agilent Technologies, Waldbronn, Germany). RNA was pooled from at least 3 mice for each treatment and genotype. The RNA was dissolved in nuclease‐free water (Invitrogen, Paysley, UK) and reverse transcribed using the Superscript III First Strand Synthesis System for RT‐PCR (Invitrogen) and oligo (dT)_12–18_ primers according to the manufacturer's specifications. The cDNA was used for qRT‐PCR analysis using the QuantiTect/PrimerAssays (Qiagen GmbH, Hilden, Germany).

The cDNA were amplified by qRT‐PCR using brilliant II SYBR Green QRT‐PCR Master Mix (Stratagene–Agilent Technologies, Waldbronn, Germany). The thermal profile consisted of 1 cycle at 50°C for 2 minutes followed by 1 cycle at 95°C (2 minute), 45 cycles at 95°C (15 seconds), 60°C (1 minute). Amplification was conducted using the M×3005P QPCR System (Stratagene–Agilent Technologies). Data were analyzed using the M×3005P analysis software (Stratagene–Agilent). Relative quantity was calculated by the comparative CT method where the amount of the target gene, normalized to the reference gene and relative to a calibrator (WT expression or control without treatment) is given by 2^−∆∆CT^. For details of the oligonucleotides used for qRT‐PCR, please refer to Table 2.

**Table 2.** Oligonucleotides Used for qRT‐PCR

Name	Symbol	Detected Transcript	Company
Actin‐β	Actb	NM_007393	Qiagen GmbH, Hilden, Germany
Beta‐2 microglobulin	B2m	NM_009735	Qiagen GmbH, Hilden, Germany
Caspase‐3	Casp3	NM_009810	Qiagen GmbH, Hilden, Germany
Glyceraldehyde‐3‐phosphate dehydrogenase	Gapdh	NM_008084	Qiagen GmbH, Hilden, Germany
Growth differentiation factor‐15	Gdf‐15	NM_011819	Qiagen GmbH, Hilden, Germany
Integrin‐αM (Cd11b)	Itgam	NM_008401	Qiagen GmbH, Hilden, Germany
Interleukin‐1β	Il1b	NM_008361	Qiagen GmbH, Hilden, Germany
Interleukin‐6	Il6	NM_031168	Qiagen GmbH, Hilden, Germany
Prostaglandin‐endoperoxide synthase 2	Ptgs2	NM_011198	Qiagen GmbH, Hilden, Germany

qRT‐PCR indicates peritoneal macrophages and real‐time polymerase chain reaction.

### Animal Study and Blood Samples

At an age of 9 weeks, the offspring were fed with an adjusted‐calories cholesterol‐enriched diet (CED, TD.88137; Harlan Teklad, Madison, WI) for a period of 12 or 20 weeks. Body weight and blood samples were taken before and after the high cholesterol diet. Plasma cholesterol and triglycerides were measured via enzymatic end point method (Randox Laboratories, Crumlin, UK) and GPO‐PAP method (glycerol‐3‐phosphate oxidase‐peroxidase; Randox Laboratories), respectively, according to the manufacturer's instructions.

### Positron Emission Tomography

Dynamic positron emission tomography (PET) studies with ^18^F‐fluoro‐2‐deoxy‐d‐glucose (^18^FDG) were done in animals 24 hours before sampling. The animals were fasted for 6 hours before PET. The animals were kept in an inhalation narcosis with Sevorane (0.5 vol%; Abbott, Wiesbaden, Germany) and oxygen (flow=500 mL/min) during the PET examination. The plasma glucose level of the animals was determined using a blood glucose sensor electrode (MediSense, Waltham, Mass). A transmission scan was done for 10 minutes before tracer administration with 2 rotating germanium pin sources to obtain cross sections for attenuation correction. The injected activity was 3 to 13.2 MBq and adjusted according to the weight of the animal, which ranged from 31.9 to 66.5 g. PET data were acquired for 1 hour in list mode on an Siemens Inveon scanner (Siemens, Erlangen, Germany) using a matrix of 256×256 (pixel size 0.3882×0.3882×0.796 mm). Images were reconstructed at defined time periods after tracer administration (2×15, 8×30, 5×60, 4×120, 2×210, 7×300 seconds). The images were reconstructed iteratively using the space alternating generalized expectation maximization method (SAGE, 16 subsets, 4 iterations) applying median root prior correction and were converted to standardized uptake value (SUV) images on the basis of the formula: SUV=tissue concentration (Bq/g)/(injected dose [Bq]/body weight [g]). The individual SUV was calculated for each study as the mean of the last 2 time frames from 50 to 60 minutes post injection.

### Gel Electrophoresis and Immunoblot Analyses

Protein extraction of the protein phase mentioned earlier was performed according to the manufacturer's instructions (TriFast, PeqLab). Twenty‐five micrograms of protein extract per lane was loaded on 14% SDS‐polyacrylamide gels and transferred to PVDF membranes (Immobilon‐P; Millipore, Billerica, MA) by electroblotting. Recombinant GDF‐15 protein and antibodies are described in Table 3. Bound antibodies were detected with peroxidase‐conjugated secondary antibodies and ECL Plus Western blotting substrate system (GE Healthcare) according to the manufacturer's manual.

**Table 3.** Antibodies Used in the Study

Name	Number	Assay	Company	Dilution
Anti‐mouse β‐actin	A5441	WB	Sigma‐Aldrich, Taufkirchen, Germany	1:10 000
Rabbit anti‐mouse HSC70	ADI‐SPA‐816	WB	Enzo Life Sciences, Lörrach, Germany	1:1000
Rabbit anti‐mouse/rat GDF‐15 (against peptide sequence HRTDSGVSLQTYDDL)		WB	Dr J. Pineda Forschungszentrum Berlin Biotechnik GmbH, Berlin, Germany	1:400
Goat anti‐rabbit IgG HRP	7074	WB	Cell Signaling/NEB, Frankfurt, Germany	1:2500
Anti‐mouse TrueBlot Ig HRP	18‐8817	WB	eBioscience, Frankfurt, Germany	1:1000
Biotinylated anti‐rabbit IgG	RPN 1004	IHC	GE Healthcare, Munich, Germany	1:800
Biotinylated goat anti‐rat IgG	STAR72	IHC	AbD Serotec, Düsseldorf, Germany	1:100
Rabbit anti‐mouse APG5/ATG5	ab 64227	IHC	Abcam, Cambridge, UK	1:500
Rat anti‐mouse CD68	MCA 1957	IHC	BD, Heidelberg, Germany	1:100
Rabbit anti‐mouse cyclooxygenase 2	ab 15191	IHC	Abcam, Cambridge, UK	1:300
Rabbit anti‐mouse IL‐6	ab 6672	IHC	Abcam, Cambridge, UK	1:1000
Rabbit anti‐mouse Ki67	ab 15580	IHC	Abcam, Cambridge, UK	1:500
Rabbit anti‐mouse macrophage migration inhibitory factor 1	ab 7207	IHC	Abcam, Cambridge, UK	1:100
Rat anti‐mouse MOMA‐2	MCA 519G	IHC	AbD Serotec, Düsseldorf, Germany	1:200
Rabbit anti‐mouse smooth muscle α‐actin	ab 5694	IHC	Abcam, Cambridge, UK	1:500
Peroxidase‐conjugated streptavidin	RPN 1051	IHC	GE Healthcare, Munich, Germany	1:500

GDF‐15 indicates growth‐differentiation factor‐15; HRP, horseradish peroxidase; IL, interleukin.

### Morphometry and Immunohistology

For morphometric and immunohistological investigations, tissue samples were taken 24 hours after PET as described earlier. The aorta ascendens, aortic arch, and the innominate artery were removed and shock‐frozen in liquid nitrogen–cooled isopentane. The extent of atherosclerotic plaques was morphometrically measured in the aorta ascendens and the innominate artery by computer‐assisted morphometry. Immunohistochemical examination of mouse innominate artery was routinely performed according to methods described earlier,^17^ using antibodies as described in Table 3.

### Statistical Analysis

The normality and variance of data were analyzed using the Shapiro–Wilk normality and the equal variance test (SigmaPlot version 12, 2010; Systat Software Inc., San José, USA), adjusted with a *P* value of 0.05 as rejection criteria. Results are presented as mean±SD. Statistical significance was calculated by the unpaired Student's *t* test, using SigmaPlot 12. In cases where the data were not normally distributed and/or the variances were not homogeneous the significance was calculated by Mann–Whitney rank sum test (SigmaPlot 12). *P* values <0.05 were considered significant.

## Results

### GDF‐15 Deficiency Affects the Expression of Inflammation or Apoptosis‐Relevant Genes in Mouse GDF‐15^−/−^/apoE^−/−^ Peritoneal Macrophages After Treatment With oxLDL

**Figure 1.**
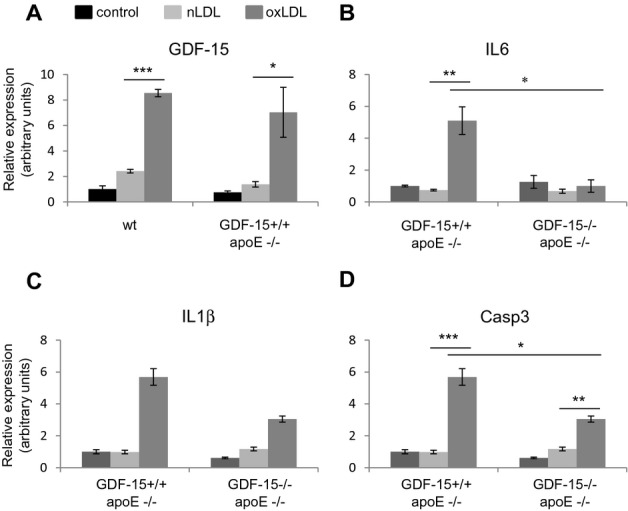
Effect of GDF‐15 deficiency on mRNA expression of apoptosis‐ or inflammation‐relevant cytokines in cultured peritoneal macrophages. Peritoneal macrophages from wild‐type (WT) (n=5), GDF‐15^+/+^/apoE^−/−^ (n=4), and GDF‐15^−/−^/apoE^−/−^ (n=5) mice were isolated, cultured, and treated with 100 μg/mL native low‐density lipoprotein (nLDL), oxidized LDL (oxLDL), or left untreated (control) for 12 hours. Cells from each group were pooled and used for RNA preparation. The mRNA levels of (A) GDF‐15, (B) IL‐6, (C) IL‐1β, and (D) caspase‐3 were determined by quantitative real‐time polymerase chain reaction (PCR); expression was normalized against β‐actin (ACTB). WT control (A) and GDF‐15^+/+^/apoE^−^/^−^ control (B–D) values were set to 1. Bars represent mean±SD of 3 experiments. *P<0.05; **P<0.01; ***P<0.005. GDF‐15 indicates growth‐differentiation factor‐15.

On stimulation with oxLDL, MФ synthesize a variety of proinflammatory cytokines and proapoptotic proteins.^20^ We used cultured peritoneal MФ from GDF‐15^−/−^/apoE^−/−^, GDF‐15^+/+^/apoE^−/−^, and WT mice to assess the effect of oxLDL on GDF‐15, IL‐6, IL‐1β, and caspase‐3 mRNA expression. MФ from WT or GDF‐15^+/+^/apoE^−/−^ mice displayed 8‐fold or, respectively, 9‐fold increased GDF‐15 expression levels (Figure 1A). Notably, oxLDL induced the expression of the proinflammatory cytokine, IL‐6 (5‐fold) in peritoneal MФ of GDF‐15^+/+^/apoE^−/−^, but not in GDF‐15^−/−^/apoE^−/−^ mice (Figure 1B). IL‐1β mRNA levels were also increased in GDF‐15^+/+^/apoE^−/−^ MФ, but this effect was not significant compared with GDF‐15^−/−^/apoE^−/−^ mice (Figure 1C). To assess the effect of GDF‐15 deficiency on apoptotic cell death, we next compared the expression levels of the proapoptotic gene caspase‐3 in peritoneal MФ in presence and absence of GDF‐15. Consistent with a proapoptotic effect of GDF‐15 caspase‐3 mRNA levels where 1.9‐fold increased in GDF‐15^+/+^/apoE^−/−^ mice (Figure 1D).

**Figure 2.**
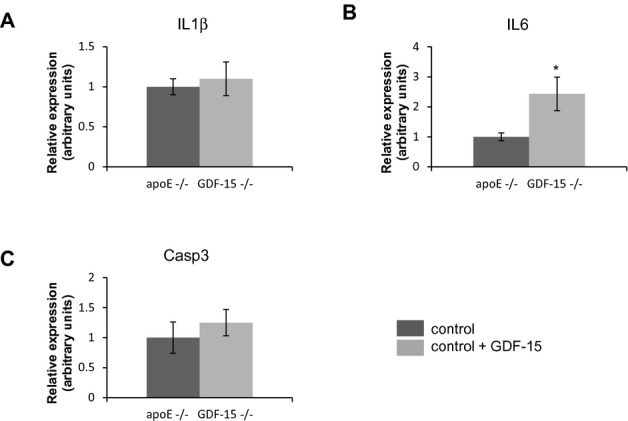
Effect of exogenous GDF‐15 on mRNA expression of apoptosis‐ or inflammation‐relevant cytokines in cultured peritoneal macrophages. Peritoneal macrophages from GDF‐15^−/−^/apolipoprotein (apo)E^−/−^ mice (n=5) were isolated, cultured, and pre‐treated with 20 ng/mL recombinant GDF‐15 (R&D Systems, Wiesbaden–Nordenstadt, Germany) or left untreated (control) for 12 hours and afterwards treated with oxLDL (12 h). Cells from each group were pooled and used for RNA preparation. The mRNA levels of (A) interleukin (IL)‐6, (B) IL‐1β, and (C) caspase‐3 were determined by quantitative real‐time polymerase chain reaction (PCR); expression was normalized against β‐actin (ACTB). Control values were set to 1. Bars represent means±SD of 3 experiments. *P<0.05; **P<0.01; ***P<0.005. GDF‐15 indicates growth‐differentiation factor‐15.

Incubation of GDF‐15^−/−^/apoE^−/−^ derived peritoneal MФ with exogenous GDF‐15 and oxLDL induced a significant increase of IL‐6 expression, whereas IL‐1β and caspase‐3 transcripts remained unaltered in this experimental setting (Figure 2). These findings argue in favor of a direct effect of GDF‐15 on IL‐6 expression, whereas the observed reduction in oxLDL induced IL‐1β and caspase‐3 expression are indirectly affected by GDF‐15 deficiency.

### GDF‐15 Is Upregulated in the Atherosclerotic Vessel Wall

**Figure 3.**
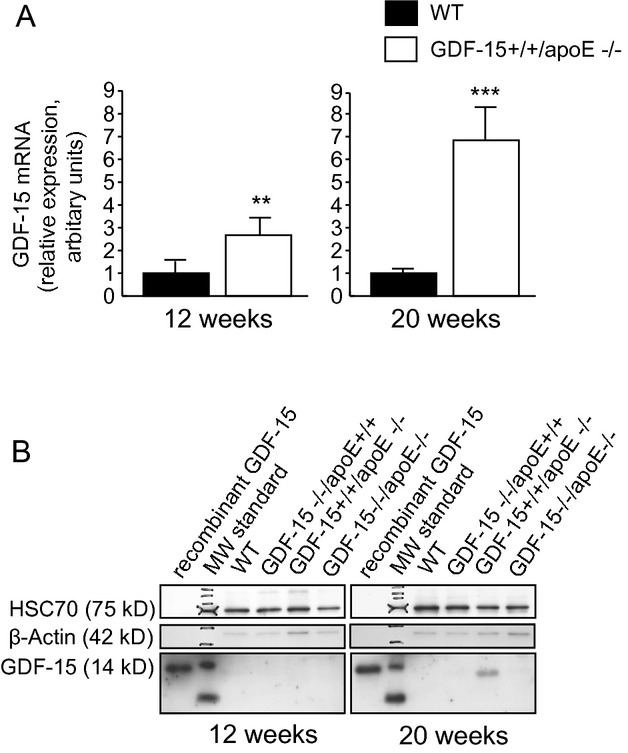
Effect of apolipoprotein (apo)E deficiency on GDF‐15 mRNA expression and protein level in the aortic arch and the innominate artery. (A) GDF‐15 mRNA expression was determined by real‐time quantitative polymerase chain reaction ( PCR) and normalized against B2m, β‐actin, and GAPDH (Table 2) in mice maintained 12 or 20 weeks on a cholesterol‐rich diet. Bars represent mean and SD of experiments using 5 mice of each group. **P<0.01; ***P<0.005 (*t* test). (B) Cell lysates from wild‐type (WT), GDF‐15^−/−^/apoE^+/+^, GDF‐15^+/+^/apoE^−/−^, and GDF‐15^−/−^/apoE^−/−^ mice fed a cholesterol‐rich diet for 12 or 20 weeks subjected to Western blot analyses with anti‐HSC70, anti‐β‐actin, or anti‐GDF‐15. The 150‐pg his‐tagged recombinant GDF‐15 protein was loaded as positive control. Data represent mean±SD of 5 mice of each genotype. *P<0.05; **P<0.01; ***P<0.005. GDF‐15 indicates growth‐differentiation factor‐15.

We have shown previously that GDF‐15 is expressed in MФ of human atherosclerotic lesions.^17^ We therefore started to determine whether GDF‐15 mRNA and protein levels are affected in experimental atherosclerosis. WT and GDF‐15^+/+^ apoE^−/−^ male mice were maintained on a CED for 12 or 20 weeks starting at an average age of 9 weeks. Figure 3A and 3B show the GDF‐15 mRNA expression and protein levels in the aortic arch including the innominate artery. Normalized to the low expression levels in WT mice, GDF‐15 mRNA was 2.6‐fold (12 weeks) and 6.8‐fold (20 weeks) increased in apoE^−/−^ mice (Figure 3A). Consistently, GDF‐15 protein immunoreactivity was exclusively detected in apoE‐deficient mice after 20 weeks on CED (Figure 3B). Together, these results suggest that development of atherosclerotic lesions is paralleled by a significant increase in GDF‐15 expression on mRNA and protein levels.

### GDF‐15–Deficient Mice Exhibit Increased Body Weight and Blood Triglyceride/Cholesterol Levels

Because of the increase in GDF‐15 levels in atherosclerotic vessels of apoE^−/−^ mice after CED, we investigated whether GDF‐15 affects obesity and blood lipid concentration using GDF‐15^−/−^/apoE^−/−^ male mice generated from heterozygous matings. WT, GDF‐15^−/−^/apoE^+/+^, GDF‐15^+/+^/apoE^−/−^, and GDF‐15^−/−^/apoE^−/−^ mice were maintained on CED for 12 or 20 weeks (Table 4). At the beginning, 9 weeks old WT and GDF‐15^−/−^/apoE^+/+^ mice revealed similar body weights. However, consistent with our previous observation that adult mice lacking GDF‐15 show an increased body weight,^20^ after 20 weeks of CED body weight of GDF‐15^−/−^/apoE^+/+^ mice was significantly increased by 10% compared with WT (GDF‐15^−/−^/apoE^+/+^: 54 g vs WT: 49 g). This difference was even more pronounced in animals with an apoE^−/−^ background (Table 4). The body weight of double knockout mice was already 8% higher at 9 weeks of age prior to CED (GDF‐15^+/+^/apoE^−/−^: 24 g vs GDF‐15^−/−^/apoE^−/−^: 26 g). After feeding CED for 12 and 20 weeks, the difference in body weight between GDF‐15^−/−^/apoE^−/−^ and GDF‐15^+/+^/apoE^−/−^ mice further increased to 18% and 35%, respectively (GDF‐15^−/−^/apoE^−/−^: 46 g/58 g vs GDF‐15^+/+^/apoE^−/−^: 39 g/43 g; Table 4).

**Table 4.** Effect of GDF‐15 Deficiency on Body Weight, Plasma Triglyceride, and Cholesterol Levels

	Apolipoprotein E^+/+^		
	GDF‐15^+/+^ (n)	GDF‐15^−/−^ (n)	*P* Value
Body weight, g			
Before CED	24±2 (19)	23±2 (20)	
After 12 wk CED	40±4 (10)	42±6 (10)	
After 20 wk CED	49±2 (9)	54±3 (10)	0.002
Triglyceride, mg/dL			
Before CED	80±32 (20)	86±31 (20)	
After 12 wk CED	66±29 (10)	101±44 (10)	0.048
After 20 wk CED	70±16 (10)	95±30 (10)	0.042
Cholesterol, mg/dL			
Before CED	102±14 (20)	83±12 (20)	<0.001
After 12 wk CED	204±25 (10)	210±73 (10)	
After 20 wk CED	304±45 (10)	309±68 (10)	

	apoE^−/−^		
	GDF‐15^+/+^	GDF‐15^−/−^	*P* Value
Body weight, g			
Before CED	24±2 (21)	26±2 (21)	0.036
After 12 wk CED	39±4 (10)	46±5 (10)	0.005
After 20 wk CED	43±4 (11)	58±4 (11)	<0.001
Triglyceride, mg/dL			
Before CED	151±90 (21)	200±94 (21)	
After 12 wk CED	185±64 (10)	233±66 (10)	
After 20 wk CED	179±25 (11)	352±167 (11)	0.01
Cholesterol, mg/dL			
Before CED	233±60 (21)	284±78 (21)	0.019
After 12 weeks CED	565±77 (10)	755±122 (10)	<0.001
After 20 weeks CED	620±57 (10)	705±153 (10)	

GDF‐15 indicates growth differentiation factor‐15; CED, cholesterol‐enriched diet.

To assess whether gain of weight in the absence of GDF‐15 correlates with changes in lipid metabolism, plasma triglyceride and total cholesterol levels were determined. However, CED of 12 and 20 weeks, significantly increased triglyceride levels in GDF‐15^−/−^/apoE^+/+^ mice by 53% and 36% compared with WT mice (GDF‐15^−/−^/apoE^+/+^: 101 and 95 mg/dL vs WT: 66 and 70 mg/dL; Table 4). Loss of GDF‐15 in apoE‐deficient mice led to a 97% higher triglyceride concentration 20 weeks after CED (GDF‐15^−/−^/apoE^−/−^: 352 mg/dL vs GDF‐15^+/+^/apoE^−/−^: 179 mg/dL; Table 4). Determination of plasma cholesterol levels in WT and GDF‐15 knockout mice revealed a 19% lower cholesterol concentration before application of CED in the absence of GDF‐15 (WT: 102 mg/dL vs GDF‐15^−/−^/apoE^+/+^: 83 mg/dL). Cholesterol levels in both groups fed with CED for 12 or 20 weeks were not significantly different (Table 4). Loss of apoE per se resulted in a 2‐ to 3‐fold increase in plasma cholesterol levels in comparison to apoE‐competent mice (Table 4). apoE knockout mice lacking GDF‐15 displayed additional significantly elevated blood cholesterol levels before and after 12 weeks feeding CED (GDF‐15^−/−^/apoE^−/−^: 284 and 755 mg/dL vs GDF‐15^+/+^/apoE^−/−^: 233 and 565 mg/dL). However, at week 20, increased cholesterol levels in double knockouts were not significantly different then GDF‐15^+/+^/apoE^−/−^ (Table 4).

### GDF‐15 Loss Reduces the Development of Atherosclerotic Lesions in the Aortic Arch and the Innominate Artery of apoE^−/−^ Mice

**Figure 4.**
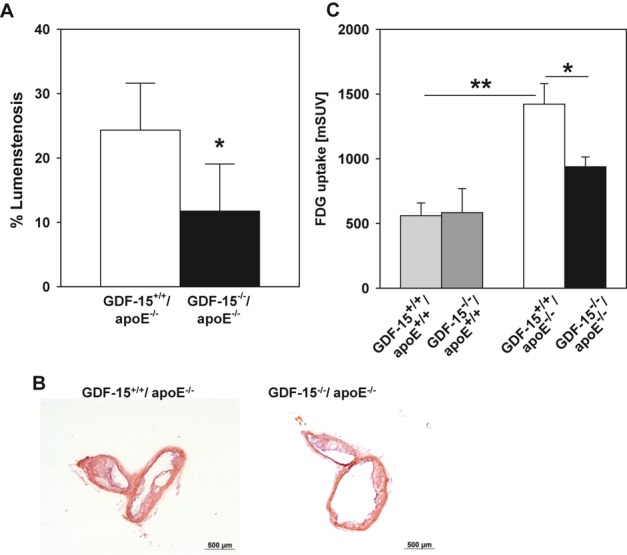
Effect of GDF‐15 deficiency on lumen stenosis and ^18^F‐fluoro‐2‐deoxy‐d‐glucose (^18^FDG) uptake in the aortic arch. After 20 weeks of CED, dynamic PET studies with ^18^FDG were performed in animals 24 hours before the aorta ascendens and aortic arch were removed and shock‐frozen in liquid nitrogen‐cooled isopentane. A, The extent of the atherosclerotic plaque was morphometrically measured in the aortic arch by computer‐assisted morphometry. GDF‐15 deficiency reduced lumen stenosis in the aortic arch by 52% (GDF‐15^−/−^/apoE‐15^−/−^: 11.8±2.6% [n=8] vs GDF‐15^+/+^/apoE‐15^−/−^: 24.3±2.6% [n=8]; *P<0.01). B, Representative hematoxylin and eosin–stained histological cross sections of the aortic arch of GDF‐15^−/−^/apoE‐15^−/−^ and GDF‐15^+/+^/apoE‐15^−/−^ mice. C, The individual standardized uptake value (SUV) was calculated for each study as the mean of the last 2 time frames from 50 to 60 minutes post infection. GDF‐15 loss significantly reduced the ^18^FDG in the aortic arch by 34% (GDF‐15^−/−^/apoE‐15^−/−^: 938.5±59.3% [n=5] vs GDF‐15^+/+^/apoE‐15^−/−^: 1422.1±112.0% [n=4]). *P<0.05; **P<0.01). GDF‐15 indicates growth‐differentiation factor‐15.

To address the question of whether loss of GDF‐15 affects the development and progression of atherosclerosis in vivo, we investigated sections of the aortic arch in GDF‐15^−/−^/apoE^−/−^ and GDF‐15^+/+^/apoE^−/−^ mice (Figure 4). After 20 weeks feeding CED, GDF‐15^−/−^/apoE^−/−^ mice showed a significantly about 52% decreased lumen stenosis in the aortic arch compared with GDF‐15^+/+^/apoE^−/−^ mice (GDF‐15^−/−^/apoE‐15^−/−^: 11.8% vs GDF‐15^+/+^/apoE‐15^−/−^: 24.3%; Figure 4A and 4B). To quantify the degree of atherosclerotic activity in the aortic arch, we analyzed GDF‐15^+/+^/apoE^−/−^ and GDF‐15^−/−^/apoE‐15^−/−^ mice using ^18^FDG PET. ^18^FDG uptake represents an index of vascular inflammation within the atherosclerotic plaque. As demonstrated in Figure 4C, GDF‐15 loss significantly reduced the ^18^FDG uptake in the aortic arch by 34%, 20 weeks after CED (GDF‐15^−/−^/apoE‐15^−/−^: 938.5 mSUV vs GDF‐15^+/+^/apoE‐15^−/−^: 1422.1 mSUV). These PET data argue for a reduction in atherosclerotic activity, consistent with the observed significantly lower lumen stenosis in the aortic arch (Figure 4A through 4C).

**Figure 5.**
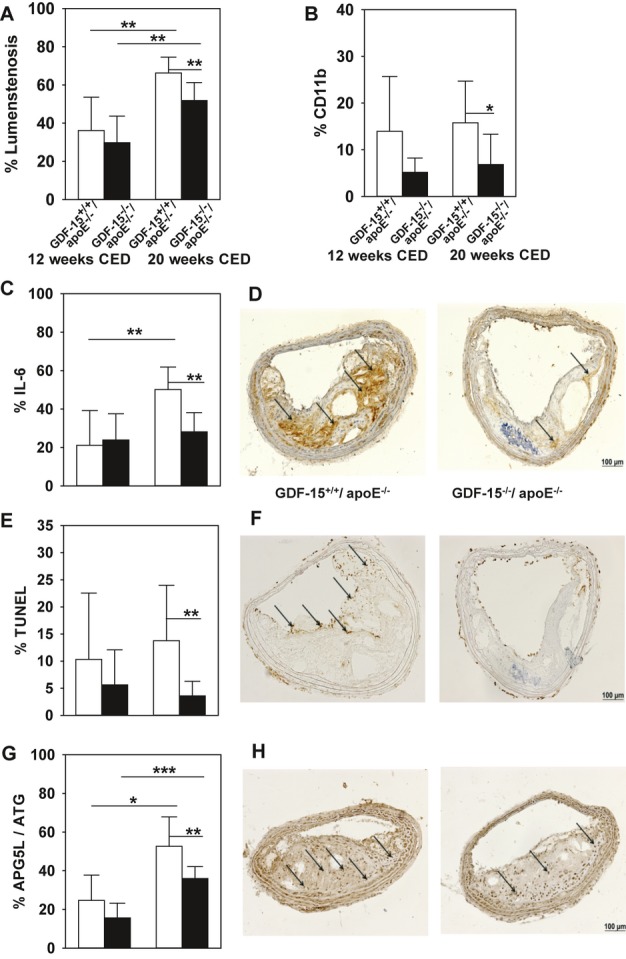
GDF‐15 deficiency reduces the development of atherosclerotic lesions in the innominate artery of apolipoprotein (apo)E^−/−^ mice. Immunohistomorphometric analyses of atherosclerotic lesions in the innominate artery of GDF‐15^+/+^/apoE^−/−^ (n=6) and GDF‐15^−/−^/apoE^−/−^ (n=5) mice after 12 and GDF‐15^+/+^/apoE^−/−^ (n=9) and GDF‐15^−/−^/apoE^−/−^ (n=11) after 20 weeks of CED. The innominate artery of GDF‐15^+/+^/apoE^−/−^ and GDF‐15^−/−^/apoE^−/−^ mice was removed and shock‐frozen in liquid nitrogen–cooled isopentane and analyzed for (A) lumen stenosis, (B) CD11b, (C, D) IL‐6, (E, F) TUNEL (TdT‐mediated dUTP‐biotin nick end labeling), and (G, H) APG5L/ATG. The data (mean±SD; *P<0.05; **P<0.01; ***P<0.001). Representative pictures of immunohistochemistry for (D) IL‐6, (F) TUNEL, and (H) APG5L/ATG. GDF‐15 indicates growth‐differentiation factor‐15; CED, cholesterol‐enriched diet.

To further analyze whether loss of GDF‐15 also affects the development of atherosclerotic plaques in other arterial vessels, we immunohistomorphometrically analyzed the innominate artery of GDF‐15^−/−^/apoE^−/−^ and GDF‐15^+/+^/apoE^−/−^ mice (Figure 5). After 12 weeks of CED, there were no significant differences in lumen stenosis between both genotypes of mice. However, consistent with the effects observed in the aortic arches of these animals, lumen stenosis in GDF‐15–deficient mice was significantly lower after 20 weeks of CED (51.9% vs 66.3%; Figure 5A).

Because we have shown previously that GDF‐15 may regulate inflammatory cell migration, differentiation, and maturation, we determined the number of inflammatory cells in atherosclerotic lesions of the innominate artery (Figure 5B and 5C). After 12 weeks feeding CED, we found a 63% reduction of the amount of CD11b^+^ cells in GDF‐15^−/−^/apoE‐15^−/−^ mice (GDF‐15^−/−^/apoE‐15^−/−^: 5.2% vs GDF‐15^+/+^/apoE^−/−^: 13.9%; Figure 5B). After 20 weeks of CED, GDF‐15^−/−^/apoE‐15^−/−^ revealed a significant 57% reduction of CD11b^+^ cells compared with GDF‐15^+/+^/apoE^−/−^ (6.8% vs 15.8%; Figure 5B).

The percentage of IL‐6 immunoreactive cells in atherosclerotic lesions of apoE‐deficient mice was not affected by the absence of GDF‐15 after 12 weeks of CED (Figure 5C and 5D). However, while the IL‐6 immunoreactive area more than doubled from 12 to 20 weeks in GDF‐15^+/+^/apoE^−/−^ mice, there was only a marginal increase in GDF‐15^−/−^/apoE^−/−^ mice.

Our previous studies demonstrated that the induction of GDF‐15 expression by oxLDL coincides with an increased rate of apoptosis in MФ.^17^ Since we found a significantly reduced lumen stenosis in GDF‐15^−/−^/apoE^−/−^ mice after 20 weeks of feeding CED, we analyzed whether the reduction might be due to changes in the rate of apoptosis. Using the TdT‐mediated dUTP‐biotin nick end labeling (TUNEL) technique, we observed 45% less TUNEL‐positive cells after 12 weeks of CED in plaques of GDF‐15^−/−^/apoE‐^−/−^ (5.6%) compared with GDF‐15^+/+^/apoE^−/−^ mice (10.3%; Figure 5E). This difference was further increased to 74% after 20 weeks (3.6% vs 13.8%; Figure 5E and 5F). Because both apoptosis and autophagy are involved in cell death processes, we additionally used antibodies directed against the apoptosis specific protein APG5L/ATG, which is required for autophagy. Loss of GDF‐15 had no effect on APG5L/ATG^+^ cells in atherosclerotic lesions after 12 weeks of CED. However, there was a significant reduction in the number of APG5L/ATG^+^ cells after 20 weeks of CED (36.0% vs 52.7%; Figure 5G and 5H). Taken together, these analyses suggest a downregulation of apoptotic and autophagic cell death in the absence of GDF‐15.

**Figure 6.**
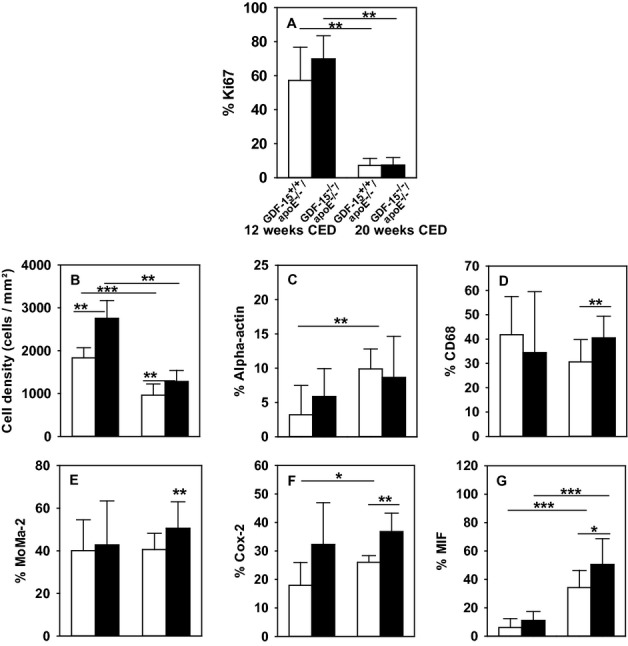
Effect of GDF‐15 on the progression of atherosclerotic plaque in the innominate artery. Immunohistomorphometric analyses of atherosclerotic lesions in the innominate artery of GDF‐15^+/+^/apolipoprotein (apo)E^−/−^ (n=6) and GDF‐15^−/−^/apoE^−/−^ (n=5) mice after 12 and GDF‐15^+/+^/apoE^−/−^ (n=9) and GDF‐15^−/−^/apoE^−/−^ (n=11) after 20 weeks of CED. Data given as mean±SD; *P<0.05; **P<0.01; ***P<0.001). Analyses of proliferation marker Ki67 (A), cell density (B), smooth muscle α‐actin (C), CD68 (D), and MoMa‐2 (E), as well as inflammation markers cyclooxygenase (COX) ‐2 (F) and MIF (G) on cryosections of the innominate artery. GDF‐15 indicates growth‐differentiation factor‐15; CED, cholesterol‐enriched diet.

To address the question of whether inhibition of development and progression of atherosclerotic lesions on loss of GDF‐15 could rely on changes in cell proliferation, we quantified the number of Ki67^+^ cells in atherosclerotic plaques (Figure 6A). In both groups (GDF‐15^−/−^/apoE^−/−^ and GDF‐15^+/+^/apoE^−/−^), long‐term feeding of CED (20 weeks) resulted in a significant decrease of Ki67^+^ cells (87% and 89%). However, there was no difference in numbers of proliferative cells between the 2 genotypes.

Having shown that GDF‐15 deficiency led to reduced cell death, we next investigated the cellularity of the atherosclerotic lesions (Figure 6B). At week 12 after CED, cell density in lesions of GDF‐15^−/−^/apoE^−/−^ mice was significantly about 50% higher compared with GDF‐15^+/+^/apoE^−/−^ mice (2754.1 cells/mm^2^ vs 1832.8 cells/mm^2^). An additional 8 weeks of feeding CED resulted in a clear decrease in the cell density in both groups (47% and 54%). Despite this reduction, the cell density was still significant higher (33%) in double knockout mice (1280 cells/mm^2^ vs 963.8 cells/mm^2^; Figure 6B).

Based on these observations, we immunohistomorphometrically measured the amount of α‐actin‐–positive smooth muscle cells, MФ, and inflammatory cells in plaques. The percentage of α‐actin–positive smooth muscle cells significantly increased in GDF‐15^−/−^/apoE^−/−^ and GDF‐15^+/+^/apoE^−/−^ between weeks 12 and 20 of CED. However, there was no difference in the percentage of α‐actin–positive smooth muscle cells in both groups (Figure 6C).

As atherosclerotic plaques containing a high number of MФ are less stable, we determined the percentage of MФ in atherosclerotic lesions of the innominate artery using CD68 and MoMa‐2 (Monocytes/Macrophages‐2) antibodies (Figure 6D and 6E). After 12 weeks of CED, we found no quantitative differences between GDF‐15^−/−^/apoE^−/−^ and GDF‐15^+/+^/apoE^−/−^ mice for both markers. Long‐term feeding led to a significantly higher percentage of CD68^+^ and MoMa‐2^+^ MФ in plaques of GDF‐15 deficient mice (CD68^+^: 40.5% vs 30.6%; MoMa‐2^+^: 50.6% vs 40.6%). These data suggest that GDF‐15 regulates the stability of atherosclerotic plaques.

Cyclooxygenase (COX)‐2 and MIF are indicators of inflammatory processes and regulate development and progression of atherosclerosis. We immunohistomorphometrically analyzed these 2 inflammatory proteins in atherosclerotic lesions of the innominate artery of both mice strains (Figure 6F and 6G). After 12 weeks of CED, the percentage of inflammatory COX‐2^+^ and MIF^+^ cells was already increased in GDF‐15^−/−^/apoE^−/−^ mice (COX‐2^+^: 32.2% vs 17.9%; MIF^+^: 11.1% vs 6%) and significantly higher after 20 weeks (COX‐2^+^: 36.8% vs 26%; MIF^+^: 50.5% vs 34.2%), suggesting that loss of GDF‐15 increases the number of COX‐2^+^ and MIF^+^ cells in atherosclerotic lesions resulting in the observed antiatherosclerotic effect.

## Discussion

Studies in animals and humans have shown that hypercholesterolemia causes infiltration and retention of LDL in the arterial intima and initiates an inflammatory response in the artery wall.^21–22^ After monocytes have attached and roll on the endothelium, they transmigrate into the subendothelial space, differentiate into MФ, and become activated due to LDL/oxLDL uptake. These cells then produce inflammatory cytokines, proteases, and cytotoxic oxygen/peroxynitrite radical molecules and activate proapoptotic signaling cascades.^23,19,24^ MФ that are stimulated with oxLDL secrete a variety of proinflammatory cytokines,^20^ implicating that the balance between inflammatory and antiinflammatory activity controls the progression of atherosclerosis.

We have demonstrated previously that GDF‐15 is inducible by oxLDL and proinflammatory cytokines in human MФ and may contribute to oxidative stress–dependent modulation of apoptosis and inflammatory processes in atherosclerotic plaques.^17^ Recent studies support this notion by showing that GDF‐15 expression levels are substantially increased in patients with cardiovascular disease^25^ and, vice versa, that GDF‐15 deficiency of leukocytes in LDL knockout mice protects against atherosclerosis.^26^ Considering that antiangiogenic stress induces GDF‐15 expression in endothelial cells,^27^ and triglyceride‐rich lipoproteins upregulate GDF‐15 by >5‐fold in human smooth muscle cells of coronary arteries,^28^ MФ are not the only source for GDF‐15 under pathological conditions. Therefore, we used a complete GDF‐15 knockout mouse developed in our laboratory and crossbred it with apoE^−/−^ mice, to study putative functions of GDF‐15 in atherosclerosis.

Our present data demonstrate that long‐term maintenance of apoE^−/−^ mice on CED is accompanied by upregulation of GDF‐15 in atherosclerotic lesions. These findings are consistent with most recent data, showing that GDF‐15 expression is increased in common iliac artery of monkeys, which have been fed a diet containing fat and cholesterol at levels comparable with those consumed in Western populations.^29^ Vice versa, we show here that lifetime lack of GDF‐15 considerably inhibits lumen stenosis and ^18^FDG uptake in experimental atherosclerosis. Because inflammatory lesions are known to show an increase in glucose metabolism, this finding indicates less inflammation in the plaques, which coincides with our data of a decrease in the number of IL‐6^+^ cells in atherosclerotic lesions of GDF‐15^−/−^/apoE^−/−^ mice. Thus, increased expression levels of GDF‐15 obviously parallel disease progression and are detrimental.

Consistent with the observation that mice overexpressing GDF‐15 show hypophagia and reduced body weight,^16^ the body weight of adult GDF‐15^−/−^ mice was significantly increased independent of the apoE genotype. In accordance with aggravated obesity, cholesterol and triglyceride concentrations were also significantly elevated in mice lacking GDF‐15. Because hypercholesterolemia and subsequent increase in triglyceride levels are associated with an increased risk of atherosclerosis, we conclude that the inhibition of lesion progression in the absence of GDF‐15 cannot be due to a regulation of plasma lipid levels. In this context it would be of interest for future studies to address the issue of whether GDF‐15 overexpressing mice reveal an enhanced lesion development and progression in experimental atherosclerosis despite their lower body weight.

According to our previous observations in human MФ,^17^ peritoneal MФ display an increase of GDF‐15 and proinflammatory cytokine IL‐6 expression after treatment with oxLDL. Interestingly, we observed that peritoneal MФ of GDF‐15^−/−^/apoE^−/−^ mice do not show such an increase after stimulation with oxLDL in comparison to GDF‐15^+/+^/apoE^−/−^. However, this effect can be restored by incubation of double knockout cells with exogenous GDF‐15, suggesting that GDF‐15 deficiency is directly associated with an inhibition of oxLDL‐induced IL‐6 expression in these MФ. An additional important finding from our studies is that, beside reduced lumen stenosis and ^18^FDG uptake in GDF‐15^−/−^/apoE^−/−^ mice, numbers of IL‐6^+^ cells in atherosclerotic lesions of the innominate artery are significantly reduced. In this context IL‐6 has been shown to exert proinflammatory effects including induction of acute‐phase proteins that may affect the plaque's fibrous cap composition.^30–31^ Other in vivo analyses further demonstrated that exogenously administered IL‐6 enhances plaque development in apoE^−/−^ mice, also owing to the upregulation of other proinflammatory factors such as TNF‐α and IL‐1.^32^ Therefore, we conclude that diminished IL‐6 expression in peritoneal MФ of GDF‐15^−/−^/apoE^−/−^ mice after oxLDL stimulation and reduction of IL‐6^+^ cells in atherosclerotic lesions of GDF‐15^−/−^/apoE^−/−^ mice may be responsible for the inhibition of the development and progression of atherosclerotic lesions in vivo. Findings corroborating this assumption are that (1) the proatherogenic role of IL‐1β and/or IL‐6 have been previously demonstrated in apoE^−/−^ mice,^33^ (2) IL‐6 mRNA and protein are expressed in the aorta of atherosclerotic apoE^−/−^ mice and correlate with the extent and size of the plaques,^34^ and (3) administration of supraphysiological concentrations of exogenous IL‐6 in the murine apoE^−/−^ deficient model of atherosclerosis dramatically enhances atherosclerotic lesion formation. Thus, our data and previous results from other groups^35^ suggest a pivotal role for IL‐6 in plaque progression. To address the question whether GDF‐15 signals though inflammatory or apoptotic pathways, we investigated the following additional molecules: Ptgs1, Ptgs2, FADD, IL‐10, CD36, Casp7, Pim2, TNF, STAB 1, STAB 2, IL‐18, Bcl10, MapK, Birc5, SR‐A, Card6, Dapk1, Fas, interferon‐γ, and SOD2 (data not shown). GDF‐15 deficiency did not affect mRNA expression of any of these genes.

Furthermore, the balance between MФ survival and death is an important determinant of lesion development and progression. In this context, we have previously demonstrated that oxLDL induces GDF‐15 expression followed by apoptosis in human MФ.^17^ We show here that GDF‐15 deficiency results in the inhibition of proapoptotic and the induction of antiapoptotic genes in peritoneal MФ after oxLDL exposition in vitro. These data are in accordance with our in vivo findings showing a reduction in TUNEL‐ or APG5L/ATG‐positive cells in atherosclerotic lesions of double knockout mice confirming the assumption that GDF‐15 may be important in MФ death in atherosclerotic lesions as has been postulated by others.^36,38^ Indeed, inhibition of autophagy, evaluated by APG5L/ATG, seems to parallel the (partly significant) inhibition of plaque development (as indicated by lumen stenosis) after 12 or 20 weeks of CED. Therefore, significant reduction in percentage of CD11b, IL‐6, TUNEL, or APG5L/ATG cells, which are in particular seen after 20 weeks of CED, seem to be responsible for the inhibition of atherosclerosis progression after 20 weeks of CED. Here we show for the first time that GDF‐15 deficiency results in inhibition of atherosclerosis progression in hypercholesterolemic mice despite an inhibition of apoptotic processes and an increase in cell density in atherosclerotic lesions. This implicates that inhibition of apoptosis is antiatherogenic and may be a therapeutic strategy to control plaque progression. In detail, the diminished progression of atherosclerosis, indicated by decreased lumen stenosis in GDF‐15^−/−^/apoE^−/−^ mice, may be the consequence of alterations in apoptotic processes, proliferation, and number of MФ or SMC in atherosclerotic lesions. In the present study, the increased cell density in atherosclerotic lesions of the innominate artery of GDF‐15^−/−^/apoE^−/−^ mice is due to an increased number of MФ (CD68^+^, MoMa‐2^+^) rather than to alterations in the number of smooth muscle cells or cell proliferation, suggesting that GDF‐15 is involved in orchestrating atherosclerotic lesion progression. In contrast to the increase of CD68^+^ and MoMa‐2^+^ in GDF‐15^−/−^/apoE^−/−^ mice, the number of CD11b‐positive cells is significantly reduced. These observations indicate that this reduction may for the most part contribute to the inhibition of atherosclerotic lesion progression. In this context, it is interesting to mention that LDL has been shown to stimulate CD11b expression in monocytes.^38^ Moreover, downregulation of these cells in obese animals significantly increases inflammation^39^ and CD11b is upregulated on monocytes after adipose tissue transplantation in addition with a dominant expression of the inflammatory cytokine IL‐6.^40^ In contrast, the number of leukocytes expressing proinflammatory cytokines COX‐2 and MIF is elevated in atherosclerotic lesions. These data suggest that, independent of increased serum lipid levels, GDF‐15 may—to a certain extent—positively regulate CD11b and inversely regulate COX‐2 and MIF. Indeed, drug experiments using, for example, NSAIDs, revealed that GDF‐15 and COX‐2 expression are inversely regulated.^41^ Furthermore, our data support the notion that increased numbers of COX‐2^+^ cells in atherosclerotic lesions of GDF‐15^−/−^/apoE^−/−^ may play an antiatherosclerotic role, as already supposed by others.^42^ Conversely, it has been demonstrated that COX‐2 deficiency results in a marked elevation of proinflammatory cytokines like IL‐6.^42^ Our data suggest that IL‐6 plays a major role in the progression of atherosclerosis and is a putative target of GDF‐15. We and others have shown that under physiological conditions, GDF‐15 is—if at all—only expressed at (very) low levels in, for example, human macrophages^10,17,19^ or in the vessel wall.^29^ Moreover, GDF‐15 expression is induced in vitro and in vivo under (oxidative) stress conditions, such as incubation of human macrophages with retinoic acid/phorbol ester^10^ or with oxLDL, C6‐ceramide, TNF‐α, or H_2_O_2_,^17^ whereby the last 3 substances have been shown to act as mediators of mechanisms relating to signal transduction activated by oxLDL.^19^ In this context, we show here that GDF‐15 is upregulated in the atherosclerotic vessel wall and most recent reports reveal evidence that GDF‐15 is associated with infarct size in experimental heart attack models and involved in the immigration of leukocytes into injured tissue.^43^ Thus, GDF‐15 may be a novel powerful biomarker for cardiovascular diseases such as heart attack or atherosclerosis. This hypothesis is further supported by recent studies on the association of GDF‐15 and coronary diseases as published by the Dallas Heart Study.^44^ Moreover, our findings and the data recently published^27^ on the functional proatherogenic role of GDF‐15 in lesion progression indicate that beside other TGF‐β superfamily members such as TGF‐β1 and BMP,^45–46^ interference with GDF‐15 may be a useful novel strategy for therapeutical intervention.
